# Secular Trends in Menarcheal Age in India-Evidence from the Indian Human Development Survey

**DOI:** 10.1371/journal.pone.0111027

**Published:** 2014-11-04

**Authors:** Praveen Kumar Pathak, Niharika Tripathi, S. V. Subramanian

**Affiliations:** 1 Department of Geography, Delhi School of Economics, University of Delhi, Delhi, India; 2 International Institute for Population Sciences, Mumbai, Maharashtra, India; 3 Department of Society, Human Development and Health, Harvard School of Public Health, Boston, Massachusetts, United States of America; Iran University of Medical Sciences, Islamic Republic of Iran

## Abstract

**Background:**

Evidence from a number of countries in Europe and North America point towards the secular declining trend in menarcheal age with considerable spatial variations over the past two centuries. Similar trends were reported in several developing countries from Asia, Africa and Latin America. However, data corroborating any secular trend in the menarcheal age of the Indian population remained sparse and inadequately verified.

**Methods:**

We examined secular trends, regional heterogeneity and association of socioeconomic, anthropometric and contextual factors with menarcheal age among ever-married women (15–49 years) in India. Using the pseudo cohort data approach, we fit multiple linear regression models to estimate secular trends in menarcheal age of 91394 ever-married women using the Indian Human Development Survey.

**Results:**

The mean age at menarche among Indian women was 13.76 years (95 % CI: 13.75, 13.77) in 2005. It declined by three months from 13.83 years (95% CI: 13.81, 13.85) among women born prior to 1955–1964, to nearly 13.62 years (95% CI: 13.58, 13.67) among women born during late 1985–1989. However, these aggregate national figures mask extensive spatial heterogeneity as mean age at menarche varied from 15.0 years in Himachal Pradesh during 1955–1964 (95% CI: 14.89–15.11) to about 12.1 years in Assam (95% CI: 11.63–12.56) during 1985–1989.

**Conclusion:**

The regression analysis established a reduction of nearly one month per decade, suggesting a secular decline in age at menarche among Indian women. Notably, the menarcheal age was significantly associated with the area of residence, geographic region, linguistic groups, educational attainment, wealth status, caste and religious affiliations among Indian women.

## Introduction

The age at menarche-the onset of first menses/periods- that heralds sexual maturation and passage from childhood to adolescence among women demonstrated a secular declining trend over the past two centuries across the globe [1]–[11]. However, enormous spatial variations in age at menarche were documented both between and within sub-national human populations. For instance, in developing countries, the mean menarcheal age varied from 16.2 years in Nepal, 15.8 in Bangladesh, 14.3 in India (Punjab), 13.5 in Sri Lanka to 12.38 in China, while in the industrialized world, it ranged from 13.3 years in Great Britain, 13.05 in France to 12.8 in the United States [12]. While the observed decline in age at menarche has flattened in many industrialized countries in Europe and North America, it has continued to drop in some countries from the developing world [13]–[20]. A few studies have already flagged uncertainties about diminishing trends in menarcheal age, particularly about the uncertain magnitude of any such decline owing to restricted sample size for specific subgroups of population [21]–[22].

Previous epidemiological evidence hinted at psychosocial and public health challenges stemming from the secular decline in age at menarche. Studies have noted that early age at menarche was strongly associated with early marriage and premature parenthood, obesity, breast cancer, ovarian cancer, psychological disorders (stress, anxiety, and depression), metabolic syndrome (diabetes, coronary heart disease, stroke and respiratory problems), substance abuse and delinquent behaviour, poor academic performance and so on [10], [23], [24]. Studies have also focused on serious societal challenges that have arisen out of the growing mismatch between early menarche induced biological maturation and subsequent psychosocial maturation [25]. These findings clearly underscore the wider public health concern emanating from the secular declining trend in age at menarche, particularly in developing countries undergoing rapid demographic, socioeconomic and nutritional transitions [26]–[27].

The age at menarche essentially has been a function of interplay between genetic variability, overall health/hygiene conditions, nutritional status and environmental influences across human population in any given territory over time [28]–[33]. However, owing to commendable improvements in the field of sanitation and hygiene, nutritional supply, public health interventions and socioeconomic advancement, the menarcheal age exhibited a secular declining trend across human population of late with considerable variability [24], [30], [34].This variability in the menarcheal age across subgroups of human population follows gradients along economic status, educational attainment, racial/ethnic differences, rural-urban system of living, supply of nutritional/health services, and family size/number of siblings etc [2], [20], [35]–[37].

Several available micro scale demographic and epidemiological evidences regarding the menarcheal age of Indian women only permits conjecture about any consistent magnitude of socioeconomic and geographic variations in age at menarche over time. The estimates presented in **Table 1** summarize the levels and patterns of age at menarche among specific groups of Indian women. The mean age at menarche reportedly varied from 16.50 years to 12.43 years across various subgroups of Indian women over the past four decades [38]–[50]. Notably, most of the studies that reported age at menarche above 13 years were estimates during 1970–1990, while remaining studies that provided estimates of age at menarche below 13 years were recent studies mostly after 2000.Some of the studies provide estimates of age at menarche across various socioeconomic groups, rural vs. urban, sportswomen, ethnic/caste groups, linguistic groups etc. For instance, a study reported the estimated age at menarche for non-poor and poor girls (13.50±0.03SD and 13.94±0.09SDrespectively) in Tirupati city of Andhra Pradesh in South India [51]. Substantial caste wise differential in age at menarche (Brahmin: 12.58±1.03SD; Maratha: 12.60±0.86SD; Scheduled Caste: 13.16±1.14SD; Other castes: 13.08±0.97SD) were also found among girls in a province in Maharashtra, Western India [52]. Some studies have shown marked differences in age at menarche among girls by socioeconomic status (Class I: 13.05±1.09SD; Class IV–V: 12.07±1.77SD), body mass index (underweight: 12.72±1.18SD; normal: 12.67±1.37SD; obese: 11.97±1.68SD) and place of residence (rural: 12.51±1.55SD; urban: 12.37±1.46SD) in Lucknow district of North India [39].

**Table 1:** Studies on menarcheal age in India.

Year	Mean age at menarche (years)	Population	Location	Reference
1972	13.80	All India pooled	India	[38]
1978	13.50±0.03 (non poor),13.94±0.09 (poor)	Girls aged 10 to 18 years	Tirupati (Andhra Pradesh)	[51]
1979	14.60±0.08	Girls aged 12 to 18 years	Rural Hyderabad (Andhra Pradesh)	[39]
1983	13.30±0.70 (Plains), 15.70±1.50 (Sportswomen), 16.50±1.20 (High altitude)	Sportswomen (16 to 27 yrs.), from plains (18 to 26 yrs.) and from high altitude (16 to 32 years)	Patiala and Amritsar (sportswomen) Delhi (plains) and Chopal (high altitude)	[40]
1983	16.38±1.53	Women from Bhotia Rajput caste	Mana valley, Chamoli district (Uttar Pradesh)	[49]
1983	12.80±0.40 (Oraon),12.76±0.35 (Munda)	Oraon and Munda girls aged 9 to 16 years	Villages of Kanke Development Block of Ranchi district (Jharkhand)	[41]
1990	13.20±1.09	Sample of 709 girls	Urban Punjabi population of Chandigarh (Punjab)	[42]
1994	14.70	Rajbanshi women	North Bengal (West Bengal)	[43]
2000	12.58±1.03 (Brahmin), 12.60±0.86 (Maratha), 13.16±1.14 (Scheduled Caste), 13.08± (Others)	School girls aged 9 to 16 years (Brahmin, Maratha and Scheduled Caste)	Pune (Maharashtra)	[52]
2001	13.50	Women from lower socio-economic category	Urban slum in Delhi (Delhi)	[44]
2006	13.857±0.01 (Brahmin), 13.859±0.01 (Rajput)	Brahmin and Rajput girls aged 9 to 15 years	Rural areas (Jammu and Kashmir)	[45]
2009	12.45±0.02 (Assamese), 12.25±0.03 (Bengali)	Assamese and Bengali girls attending school aged 10 to 16 years	Guwahati (Assam)	[46]
2009	12.62±1.05	Girls 9 to 16 years from one convent and one municipal corporation school	Pune (Maharashtra)	[47]
2011	12.43±1.49	Girls aged 10 to 19 years urban and rural govt. schools	Lucknow (Uttar Pradesh)	[50]
2011	13.22±0.88 (recall), 12.13±0.79 (probit)	Adolescent girls	East Khasi hill district (Meghalaya)	[48]

However, many earlier studies [53] debated the unresolved issues of any secular declining trend in age at menarche and there was no conclusive evidence to support the above hypothesis for the overall Indian population. In most of the available scientific literature on age at menarche in the Indian context, the unit of analysis has been school girls/adolescents or sports women from a specific localized area (town/city/rural areas). Besides, most of the previous studies were confined to a limited geographic context and they used varying methodology, thereby making it exceedingly difficult to draw any befitting spatiotemporal comparisons of age at menarche in India and/or its any regional dimensions thereof.

In order to fulfil this research gap, the present study uses a nationally representative household survey data, perhaps for the first time, to estimate the magnitude of a secular trend in age at menarche among ever married women (15–49 years) in India during 1955–1989. This study employed a pseudo birth cohorts approach to estimate secular trends in age at menarche. It also highlights the observed spatial heterogeneity (both inter-regional and inter-state) in the age at menarche among ever-married women (15–49 years) during the study period. Finally, it examines the association of selected socioeconomic, demographic, anthropometric and contextual risk factors with menarcheal age among Indian women.

## Methods

### Ethical Statement

The study was based on an anonymous public use data set with no identifiable information on the survey participants; therefore no ethics statement is required for this work.

### Study Design and Data

The cross-sectional data for the present study comes from the Indian Human Development Survey (IHDS) conducted during November 2004 and October 2005 under the collaborative project of National Council of Applied Economic Research (NCAER), New Delhi and the University of Maryland. The IHDS is a nationally representative, multi-topic face-to-face survey of 41554 households, covering 215754 individuals from 1503 villages and 971 urban neighborhoods located in 384 districts spread across 33 states and union territories (excluding Andaman and Nicobar Islands and Lakshadweep) in India [54]. It provides robust indicators on various dimensions of human development including health, education, marriage and family, gender relations, fertility, family planning, and health care utilization at both national and sub-national levels. The survey questionnaires were translated into 13 Indian languages and administered through trained local field interviewers. Villages and urban blocks formed the primary sampling unit (PSU) consisting of 150–200 households from which the sampled households were randomly selected. However, urban and rural PSUs were selected using different designs. The survey response rate was 92 percent for the total [43].

The IHDS asked all ever-married women in the age group 15–49, ‘*How old were you when you first started having your periods?*’ during the face-to-face interview. Out of a total 105949 eligible sampled respondents, about 14545 women (14 percent) did not report their age at first menstrual period. Around ten women could not accurately report either their current age in completed years or year of birth. Hence, the final analytical sample was reduced to 91394 ever married women aged 15–49 born during 1955–1989.

### Outcome Variable

In IHDS, all ever married women aged 15–49 years were asked to report about the age (in completed years) at which they experienced the first menstrual period. Accordingly, we used information of age at menarche (continuous variable) among ever married women as the dependent variable for the present study.

### Exposure Variables

This study used a set of theoretically appropriate demographic, socioeconomic, anthropometric and contextual characteristics in the analyses. We used information on year of birth of ever married women 15–49 years to identify the birth cohorts (1955–1964, 1965–1974, 1975–1984,> = 1985) in order to generate the secular trends in age at menarche. However, in multivariate analyses, the information on year of birth of ever married women was used as continuous variable to test the secular trend in age at menarche.

The socioeconomic characteristics of women were accounted through female education (none, primary, secondary, senior secondary, college and above), caste affiliation (Scheduled caste, Scheduled tribe, Other Backward Classes, Other/Forward Castes), religious groups (Hindu, Muslims, Christian, Sikh, others) and wealth quintile.

Data on income or expenditure are usually not collected in large scale surveys like Demographic and Health Surveys (DHS). Thus, customarily a composite index representing the wealth status of the household is generated as proxy of economic status, using information on ownership of a set of consumer durable asset, access to utilities and infrastructure, and housing characteristics. IHDS collected information on income, and household assets, utilities and housing characteristics. However, income measures suffer from numerous limitations including measurement errors and under-reporting of actual income, particularly from higher income groups [55]. On the other hand, household possession of assets and amenities suggests accumulation over the years and hence emerges as a better indicator of the household's long-term economic status. Therefore, we used information on a range of 49 sub items representing ownership of consumer durable assets, access to utilities and infrastructure, and housing characteristics to generate a composite index representing the relative standing of the households according to their wealth status by employing principal component analysis [56]–[60]. Eigen values for each factor are depicted by scree plot **(Figure S1)**. We also tested the reliability of this composite measure using Cronbach's alpha reliability test, and the results confirmed robustness of the same. A similar approach was employed by a couple of studies in India, which analysed the same data to examine spatial disparities in maternity care, mortality burden and socioeconomic status [61]–[62].

The anthropometric status was assessed through the body mass index of women (kg/m^2^). The body mass index (BMI) was calculated as weight in kilograms divided by height in meters squared (kg/m^2^) based on values recorded from the actual measurements of both the height and weight of sampled women respectively. The World Health Organization [63] (WHO, 2004) recommended a tenfold classification of body mass index for the Asian population ranging from severe underweight (<16 kg/m^2^) to obese class-III (> =  40 kg/m^2^). Drawing from the above standard classification and a study from India [64], we categorized BMI of women into the following four groups: underweight (<18.5 kg/m^2^); normal (18.5–24.9 kg/m^2^); overweight (25.0–29.9 kg/m^2^); obese (> = 30.0 kg/m^2^; not measured) considering sufficient sample size in each category. The contextual variations were captured through area of residence (rural vs. urban), geographical regions (north, central, east, north-east, west and south) and major linguistic groups (Hindi, Bangla, Gujarati, Marathi, Kannada, Malayalam, Tamil, Telugu, other languages) in India [65].

### Statistical Analysis

We used bivariate analysis to present the age at menarche across birth cohorts, regions/provinces and salient socioeconomic, demographic, anthropometric and contextual characteristics among Indian women during the study period. We used One-way analysis of variance (ANOVA) to test the statistical differences in age at menarche across different risk factors. Considering the continuous nature of the dependent variable, we fit a multiple linear regression model to examine secular trend in age at menarche, and further to investigate the plausible association between various risk factors with menarcheal age among Indian women. We also account for the stratification, complex survey design and used appropriate weights in the analysis.

## Results

### Secular trends in menarcheal age

**Figure 1:**
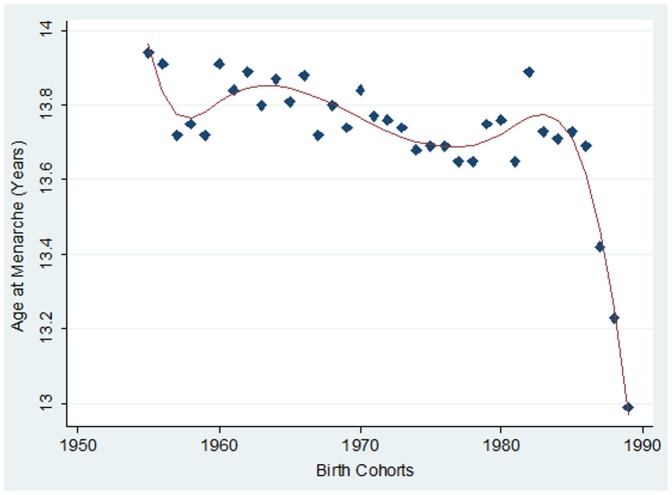
Secular trends in mean menarcheal age among ever married women in India, IHDS, 2004–2005.

**Table 2** reveals the mean age at menarche across birth cohorts among ever-married women aged 15–49 years in India. The data demonstrates that mean age at menarche was 13.76 (with a 95% CI of 13.75–13.77) among ever married women aged 15–49, whose mean age was 32.61 years. Importantly, the mean age at menarche depicted a long-term decreasing trend towards the younger birth cohorts (**Figure 1**). In fact, there was a difference of about 0.21 years in menarcheal age between the oldest and the youngest cohorts. The estimates of the mean age at menarche of recent three younger cohorts were compared to the 1955–1964 birth cohorts by performing One-way ANOVA test and were all found to be significantly different (p<0.001).

**Table 2:** Mean menarcheal age by birth cohorts among ever-married women (15-49 years) in India, IHDS, 2004–2005.

Characteristics	Mean	95% CI		P-values	Percent distribution of women	Sample Size (N)
		Lower Bound	Upper Bound			
Total	13.76	13.75	13.77		100	91394
Birth cohort						
1955–1964	13.83	13.81	13.85		23.2	21218
1965–1974	13.76	13.75	13.78		40.0	36571
1975–1984	13.72	13.71	13.73		34.0	31060
1985–1989	13.62	13.58	13.67	0.000	2.8	2545

Note: Analysis of Variance (ANOVA) test has been applied to check the difference in mean age at menarche across birth cohorts; P<0.001 indicates statistically significant at 1%; Unweighted samples have been reported; CI is confidence interval.

### Regional variations in menarcheal age

**Figure 2:**
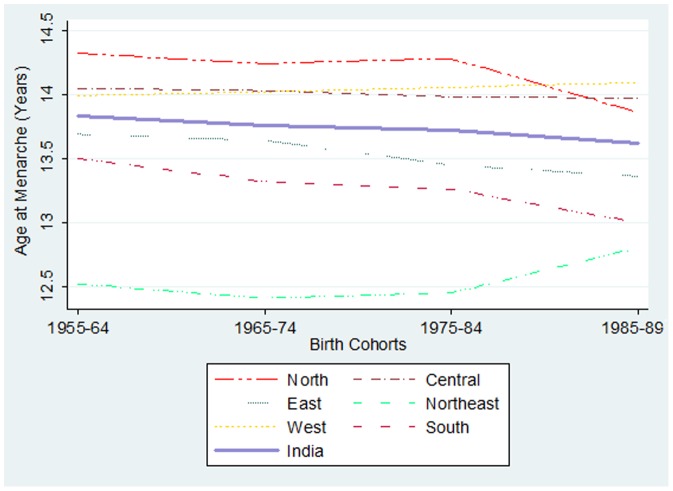
Birth Cohort specific mean age at menarche among ever married women across major geographic regions in India, IHDS, 2004–2005.

**Figure 2** demonstrates the synoptic view of the stark regional heterogeneity in cohort specific mean age at menarche among ever married women in India. The mean age at menarche was highest in the northern region (14.29, with 95% CI of 14.27–14.31) followed by the central (14.02, with 95% CI of 14.01–14.03) and western region (14.01, with 95% CI of 13.99–14.03), and it was least in the north-eastern region (12.60, with 95% CI of 12.56–12.64). The estimates of the mean age at menarche across geographic regions were compared by performing One-way ANOVA test and were found to be significantly different (p<0.001).The regional differentials in mean age at menarche remain substantially large over the study period.

**Table 3** presents detailed account of remarkable inter-regional and inter-state differentials in the mean age at menarche across birth cohorts of ever married women in India. Interestingly, most of the states from northern central and western part of India exhibit relatively higher mean age at menarche over the study period. For instance, Himachal Pradesh, Uttar Pradesh, Madhya Pradesh, Maharashtra, Rajasthan, Haryana and Punjab show evidence of consistently higher age at menarche than national average. On the contrary, many states from the north-eastern, eastern and southern parts of India demonstrate considerably lower mean age at menarche. Some noticeable among them includes Arunachal Pradesh, Assam, Andhra Pradesh, Manipur, Karnataka and Tamil Nadu where age at menarche remained consistency below the national average during the study period.

**Table 3:** Mean age at menarche by birth cohorts among ever married women (15–49 years) across geographic regions/states in India, IHDS, 2004–2005.

Birth Cohorts	1955–64			1965–74			1975–84			1985–89		
Region/State	AAM	95% CI		AAM	95% CI		AAM	95% CI		AAM	95% CI	
India	13.83	13.81	13.85	13.76	13.75	13.78	13.72	13.71	13.73	13.62	13.58	13.67
Northern India	14.32	14.28	14.36	14.24	14.21	14.27	14.28	14.25	14.31	13.86	13.76	13.96
Jammu & Kashmir	14.43	14.29	14.56	14.41	14.26	14.56	14.20	14.06	14.34	13.00	13.00	13.00
Himachal Pradesh	15.00	14.89	15.11	15.24	15.16	15.33	15.12	15.04	15.20	15.29	14.91	15.66
Punjab	14.29	14.23	14.36	14.15	14.11	14.20	14.23	14.17	14.29	13.68	13.33	14.04
Uttaranchal	14.97	14.79	15.15	14.55	14.45	14.65	14.65	14.52	14.78	14.00	14.00	14.00
Haryana	14.58	14.50	14.67	14.55	14.48	14.62	14.55	14.48	14.61	14.31	14.15	14.47
Delhi	13.49	13.35	13.62	13.75	13.64	13.87	13.58	13.46	13.70	14.23	13.66	14.81
Rajasthan	14.07	13.99	14.14	14.02	13.96	14.08	14.11	14.05	14.16	13.66	13.52	13.80
Western India	13.99	13.94	14.03	14.02	13.99	14.06	14.09	14.06	14.12	14.08	13.96	14.19
Gujarat	13.75	13.69	13.81	13.88	13.84	13.93	13.87	13.82	13.92	13.34	13.22	13.46
Maharashtra	14.13	14.07	14.19	14.10	14.06	14.15	14.21	14.16	14.25	14.45	14.31	14.58
Goa	13.49	13.21	13.77	13.57	13.24	13.90	13.34	13.13	13.56	na	na	na
Central India	14.05	14.02	14.08	14.03	14.01	14.05	13.98	13.95	14.00	13.97	13.88	14.06
Chhattisgarh	13.79	13.72	13.86	13.89	13.84	13.95	14.04	13.97	14.11	13.54	13.32	13.76
Madhya Pradesh	13.98	13.93	14.03	13.88	13.85	13.92	13.97	13.93	14.01	13.68	13.52	13.83
Uttar Pradesh	14.14	14.10	14.19	14.11	14.08	14.14	13.97	13.94	14.00	14.14	14.02	14.27
Eastern India	13.69	13.64	13.73	13.64	13.61	13.67	13.45	13.42	13.48	13.36	13.27	13.44
Bihar	13.77	13.70	13.84	13.92	13.86	13.98	13.72	13.67	13.77	13.82	13.65	13.99
Jharkhand	14.26	14.14	14.38	14.15	14.07	14.24	13.46	13.40	13.52	13.45	13.23	13.66
Orissa	13.35	13.30	13.40	13.34	13.30	13.39	13.24	13.20	13.28	13.02	12.92	13.11
West Bengal	13.49	13.41	13.57	13.26	13.20	13.31	13.28	13.21	13.35	13.09	12.92	13.25
Southern India	13.50	13.46	13.53	13.32	13.30	13.35	13.26	13.23	13.29	13.00	12.89	13.10
Andhra Pradesh	13.36	13.28	13.43	13.11	13.06	13.15	13.18	13.13	13.24	12.91	12.71	13.12
Karnataka	13.06	13.00	13.11	13.05	13.00	13.09	13.06	13.01	13.11	13.03	12.88	13.18
Kerala	13.45	13.37	13.54	13.21	13.15	13.27	13.21	13.13	13.28	13.31	13.15	13.46
Tamil Nadu	14.01	13.93	14.09	13.89	13.83	13.94	13.60	13.52	13.68	13.01	12.70	13.31
Northeast India	12.52	12.43	12.60	12.41	12.34	12.47	12.45	12.37	12.53	12.80	12.47	13.13
Arunachal Pradesh	12.12	12.02	12.23	12.29	12.20	12.38	12.32	12.14	12.49	12.00	12.00	12.00
Manipur	14.08	13.79	14.37	14.01	13.76	14.24	14.77	14.48	15.06	13.00	13.00	13.00
Mizoram	14.44	14.29	14.59	14.14	14.05	14.22	14.38	14.10	14.66	14.00	14.00	14.00
Tripura	13.44	13.27	13.61	13.46	13.34	13.58	13.26	13.05	13.47	13.92	13.54	14.29
Meghalaya	13.67	13.43	13.90	13.68	13.43	13.93	13.38	13.11	13.65	13.37	11.90	14.84
Assam	11.97	11.88	12.06	11.86	11.78	11.93	11.99	11.92	12.06	12.10	11.63	12.56
Sikkim	12.27	12.10	12.44	12.00	11.98	12.02	12.00	12.00	12.00	na	na	na
Nagaland	13.23	12.76	13.69	13.01	12.70	13.31	13.34	12.92	13.77	na	na	na

Note- AAM refers to age at menarche; na- indicates data not available.

Socioeconomic, anthropometric and contextual variations in menarcheal age.

**Figure 3:**
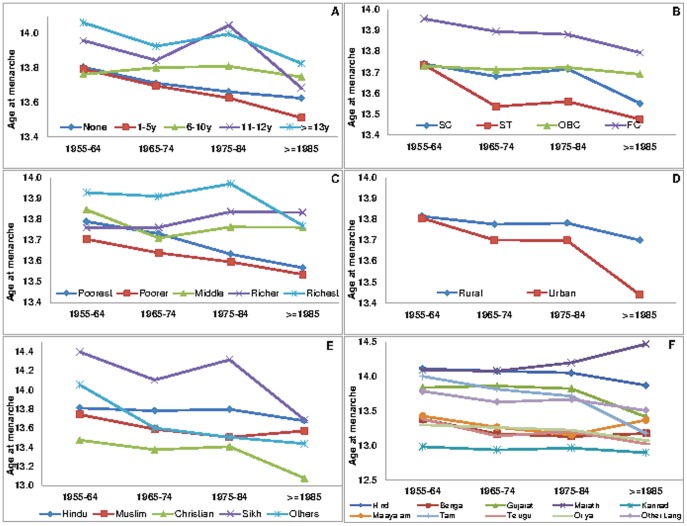
Socioeconomic and cultural patterning of mean age at menarche across birth cohorts of women in India, IHDS 2004–2005. A. Age at menarche by education across birth cohorts. B. Age at menarche by caste across birth cohorts. C. Age at menarche by wealth status across birth cohorts of women. D. Age at menarche by residence across birth cohorts of women. E. Age at menarche by religion across birth cohorts of women. F. Age at menarche by language across birth cohorts of women.

**Table 4** presents a descriptive analysis of mean age at menarche across selected socioeconomic, anthropometric and contextual characteristics among ever married women in India. The distribution of the educational profile suggests that nearly 50 percent of the respondents received no formal education. However, close to 40 percent of the respondents were educated either up to primary or secondary level and a small proportion received more than secondary education (10 percent). A group of women with no education was tested against those in the other higher educational categories and were found to have a significantly lower mean age at menarche (p<0.001) (**Figure 3**).

**Table 4:** Mean age at menarche across selected socioeconomic, anthropometric and contextual characteristics among ever-married women (15–49 years) in India, IHDS, 2004–2005.

Characteristics	Mean	95% CI		P-values	Percent distribution of women	Sample Size (N)
		Lower Bound	Upper Bound			
Years of education						
None	13.75	13.74	13.76		45.3	41383
1–5y	13.65	13.63	13.67		16.3	14887
6–10y	13.78	13.77	13.80		28.3	25841
11–12y	13.90	13.87	13.94		5.1	4651
>12y	13.98	13.94	14.02	0.000	5.1	4632
Wealth quintile						
Poorest	13.70	13.68	13.72		20.0	18282
Poorer	13.63	13.62	13.65		20.0	18280
Middle	13.76	13.74	13.78		20.0	18280
Richer	13.79	13.77	13.81		20.0	18274
Richest	13.93	13.91	13.95	0.000	20.0	18278
Caste group						
Scheduled tribe	13.64	13.61	13.67		7.8	7156
Scheduled caste	13.68	13.66	13.70		20.4	18622
Other backward class	13.79	13.78	13.80		40.1	36612
Other castes	13.81	13.79	13.82	0.000	31.7	29004
Religion						
Hindu	13.77	13.77	13.78		79.7	72814
Muslim	13.65	13.63	13.68		13.2	12078
Christian	13.45	13.40	13.50		2.6	2338
Sikh	14.17	14.13	14.21		2.6	2331
Others	13.88	13.82	13.94	0.000	2.0	1833
Body Mass Index (Kg/M^2^)						
Missing values	13.78	13.77	13.79		46	42270
<18.5	13.75	13.74	13.77		22	20482
18.5–24.9	13.74	13.72	13.76		25	22380
25.0–29.9	13.77	13.74	13.82		5.0	4608
> = 30.0	13.78	13.71	13.85	0.002	1.8	1654
Area of residence						
Rural	13.77	13.76	13.77		67	61023
Urban	13.75	13.73	13.76	0.000	33	30371
Geographic regions						
North	14.26	14.24	14.28		23	21276
West	14.04	14.02	14.06		14	12514
Central	14.02	14.00	14.03		21	18764
East	13.57	13.55	13.59		17	15321
South	13.34	13.32	13.35		22	19957
North-East	12.46	12.41	12.50	0.000	3.9	3562
Linguistic groups						
Hindi	14.02	14.01	14		47.0	42990
Bengali	13.26	13.23	13.2		5.3	4846
Gujarati	13.82	13.79	13.8		5.1	4667
Marathi	14.81	14.78	14.8		7.4	6722
Kannad	13.05	13.03	13.1		9.0	8226
Malayalam	13.27	13.23	13.3		3.6	3330
Tamil	13.82	13.78	13.9		4.0	3616
Telugu	13.19	13.16	13.2		5.0	4572
Oriya	13.29	13.27	13.3		4.9	4496
Other language	13.44	13.4	13.5	0.000	8.7	7929

Note: Analysis of Variance (ANOVA) test has been applied to check the difference in mean age at menarche across categories of covariates; P<0.001 indicates statistically significant at 1%; Unweighted samples have been reported; Percent total may not add upto 100 due to rounding off.

Profiles of interviewed women by household wealth quintile exhibit approximately equal proportions from the poorest to the richest group. However, the mean age at menarche shows an increasing trend from the poorest to richest wealth quintile and the difference between the women of the poorest wealth quintile with other categories was significant (p<0.001) (**Figure 3**).

Caste identifications reflect systematic deprivation over extended historical time periods owing to caste based hierarchical positions prevalent in Indian society. The Indian Constitution provides special privileges to historically most deprived communities like the Scheduled Caste (SC) and Scheduled Tribe (ST) groups and Other Backward Classes (OBC) to ensure their protection and participation in the mainstream [66]. The residual categories of ‘Other/Forward Castes’ group are so called socially forward social groups in the caste based hierarchical system. Data on caste affiliations of interviewed women indicate that majority of them belonged to Other Backward Classes (40 percent) followed by other castes (32 percent), Scheduled Caste (20 percent) and Scheduled Tribe (4 percent) respectively. Compared to women from the Scheduled Tribes, the mean age at menarche from disparate caste groups was significantly higher (p<0.001). Women from other/forward caste groups experienced a relatively higher mean age at menarche than their counterparts from lower castes groups (**Figure 3**).

Religious compositions of interviewed women show that the majority consisted of Hindus (79.7 percent) followed by Muslims (13.2 percent), while Christians, Sikhs and other religious communities accounted for about two percent each of the sampled population respectively. The evidence indicates significant differences in menarcheal age among women by their religious affiliations (p<0.001). Women from Christian and Muslim groups experienced a relatively early menarcheal age compared to women from Hindu, Sikh and other religious groups.

Body mass index was measured only for little more than 50 percent of the women interviewed for whom the information on either height or weight was available. Among these, about one fourth had normal BMI, one-fifth had low BMI and the remaining six percent had high BMI. Women with low BMI (BMI<18.5) were tested against those with other relatively moderate/high BMI categories and were found to have significantly different mean age at menarche (p<0.001). Women with relatively higher BMI (> = 25.0) experienced significantly elevated menarcheal age than their counterparts with low BMI (< = 18.5) (p<0.001).

The profile of interviewed women by area of residence indicates that close to67 percent of the respondents resided in rural areas, while the remaining 33 percent lived in urban centres. However, then mean age at menarche of women living in urban areas was significantly lower than that of their rural counterparts (p<0.001). The geographic composition of women indicates that majority of the respondents resided in the northern region (23 percent), followed by south (22 percent), central (21 percent), east (17 percent), west (14 percent) and north-eastern (four percent) parts of India. The menarcheal age varies significantly across geographic regions (p<0.001). Women who lived in the north-eastern region experienced menarche at a relatively early age when compared to their counterparts in other geographic regions. We also investigated the variations in menarcheal age across major linguistic groups in India. Data suggest that majority of sample women speak Hindi, followed by Kannada, Marathi, Bengali, Gujarati, Telugu, Oriya, Tamil, Malayalam and other linguistic groups. Hindi speaking women were tested against those speaking other Indian languages and were found to have a significantly different mean age at menarche (p<0.001).

We also examined the socioeconomic pattern in the mean age at menarche across the states of India (**Table S1–S4**). The results suggest a significant socioeconomic gradient in menarcheal age, as women with no/low education, Scheduled Caste/Scheduled Tribe and economically poor household experienced relatively early menarche than their better off counterparts across states in India. Further, the socioeconomic gradients in mean age at menarche were pronounced among women from the southern, eastern and north-eastern states.

### Predictors of Menarcheal Age

The results from descriptive analysis established secular declining trends in menarcheal age among ever married women in the age group 15–49 in India. These results also bring out sizeable variations in menarcheal age across socioeconomic, anthropometric and contextual attributes of women. However, in order to test whether these secular trends in menarcheal age are true, we fit a multiple linear regression model adjusting for potential socioeconomic, anthropometric and contextual risk factors. The age at menarche was the outcome variable and the year of the respondent's birth (continuous), education, wealth quintile, caste, religion, area of residence, geographic region and linguistic groups were exposure variables.

The fitted regression model had an adjusted *R*-square value of 0.138, which explained 14 percent of the total variation found in the age at menarche (see **Table 5**). The estimated regression parameter indicated that one unit increase in the year of birth was associated with an average of 0.009 year decrease in age at menarche among Indian women after adjusting for other socioeconomic, anthropometric and contextual characteristics. This confirmed the secular declining trends in menarcheal age among women in India.

**Table 5:** Multiple linear regression predicting menarcheal age among ever-married women (15–49 y) in India, IHDS, 2004–2005.

Estimated regression coefficients parameter	Coefficient	95%CI		P-value
Intercept	29.900	27.935	31.865	0.000
Year of Birth	−0.008	−0.009	−0.007	0.000
Years of education				
None^#^	0.000	0.000	0.000	-
1–5y	−0.025	−0.048	−0.003	0.025
6–10y	0.156	0.134	0.178	0.000
11–12y	0.256	0.215	0.298	0.000
>12y	0.339	0.294	0.384	0.000
Wealth Quintile				
Poorest^#^	0.000	0.000	0.000	-
Poorer	0.034	0.012	0.057	0.003
Middle	0.061	0.035	0.086	0.000
Richer	0.070	0.041	0.100	0.000
Richest	0.061	0.026	0.096	0.001
Caste group				
Scheduled caste^#^	0.000	0.000	0.000	-
Scheduled tribe	0.034	0.011	0.057	0.044
Other backward class	0.096	0.075	0.117	0.000
Other castes	0.071	0.047	0.094	0.000
Religion				
Hindu^#^	0.000	0.000	0.000	-
Muslim	−0.026	−0.051	−0.003	0.030
Christian	0.064	0.009	0.120	0.024
Sikh	−0.002	−0.074	0.071	0.959
Others	0.130	0.075	0.185	0.000
Body mass index (Kg/M^2^)				
<18.5^#^	0.000	0.000	0.000	-
18.5–24.9	0.014	−0.007	0.036	0.183
25.0–29.9	−0.038	−0.077	0.001	0.055
> = 30.0	0.043	−0.019	0.106	0.178
Missing(weight/height not measured)	0.005	−0.013	0.024	0.568
Area of Residence				
Rural^#^	0.000	0.000	0.000	-
Urban	−0.170	−0.191	−0.149	0.000
Geographic regions				
North^#^	0.000	0.000	0.000	-
Central	−0.265	−0.293	−0.237	0.000
East	−0.508	−0.540	−0.476	0.000
North-East	−1.705	−1.762	−1.648	0.000
West	0.003	−0.059	0.066	0.917
South	−1.198	−1.328	−1.067	0.000
Linguistic Groups				
Hindi^#^	0.000	0.000	0.000	-
Bangla	−0.428	−0.464	−0.392	0.000
Gujarati	−0.500	−0.568	−0.432	0.000
Marathi	−0.216	−0.281	−0.151	0.000
Kannad	−0.061	−0.195	−0.072	0.366
Malayalam	0.002	−0.135	0.139	0.975
Tamil	0.700	0.566	0.833	0.000
Telugu	0.094	−0.037	0.226	0.161
Oriya	−0.492	−0.535	−0.448	0.000
Other language	−0.176	−0.223	−0.130	0.000
*Adjusted R-Square*	0.138			
*Number of observation*	91394			

Note-^#^ Reference groups

The results also suggest a positive association between mean menarcheal age and women's education as women with greater than 12 years of education had 0.339 years higher mean age at menarche than women with no education, all else being equal. Similarly, the economic status of women was also positively associated with mean menarcheal age as women from the wealthiest quintile had 0.061 years higher mean age at menarche than the poorest wealth quintile, keeping other variables constant. The study also found statistically significant differences in mean menarcheal age across castes. Women from Other/Forward Castes had 0.071 years higher mean age at menarche than their counterparts from Scheduled Caste groups. Religion also predicted statistically significant differences in mean menarcheal age; for instance the mean menarcheal age for Muslim women were 0.026 years earlier than that of their Hindu counterparts. However, the data not support any significant association between mean menarcheal age and body mass index of women.

Interestingly, women from urban areas experienced mean age at menarche 0.170 years earlier than their counterparts from rural areas, all else being equal. These results also suggest statistically significant differences in menarcheal age among women across various geographic regions of India. Women residing in the north-eastern region had mean age at menarche 1.705 years earlier than their counterparts from the northern region. The mean menarcheal age also varied significantly across major linguistic groups in India. Women speaking Gujarati had mean age at menarche 0.500 years earlier than their Hindi speaking counterparts.

## Discussion and Conclusion

This study uses a nationally representative data that provides secular trends in menarcheal age among ever married women (15–49 years) in India during 1955–1989. It employs a pseudo-cohort approach to estimate the magnitude of secular decline in menarcheal age using a cross-sectional data. It throws light upon glaring spatial heterogeneity (both inter-regional and inter-state) in menarcheal age among Indian women over time. It also examines the association of salient socioeconomic, anthropometric and contextual risk factors associated with menarcheal age among women in India.

The results clearly establish a reduction of nearly one month per decade, suggesting a secular decline in age at menarche among Indian women during 1955–1989. These relatively modest changes occur due to improvement in the field of scientific and technological advancement, economic growth, agriculture, food supply, public health systems and hygiene, which markedly help to improve the general health, nutritional level and over all standard of living of the population of past several decades. However, these findings assume critical significance as India ranks among one of the youngest nations with the largest adolescents population (243 million) followed by China (207 million), the United States of America (44 million), Indonesia and Pakistan (41 million each) [67]. Furthermore, nearly30 percent of India's population (327 million) ranges between 10–24 years of age and approximately 70 percent of India's population age is below 35 years [68].Therefore, any mismatch between physical puberty (owing to early menarcheal age) and social puberty (age at which people are mentally, educationally and legally equipped to function as adults in modern societies) may trigger ignorant health damaging behaviour among adolescent/youth including early and unprotected sexual debut, sexually transmitted disease (STD)/reproductive tract infections (RTI) including HIV/AIDS and teenage pregnancies [69]. These inadvertent occurrences may be averted though proper planning and policies that encourage family life education and empower adolescents/youth through culturally sensitive and age appropriate skills/knowledge creation [70].

The results from this study also highlight stark spatial heterogeneity in mean menarche age among women across geographic regions and states/provinces in India over time. Women from the northern, central and western parts of India experienced a relatively higher mean age at menarche as compared to their counterparts from the north-east, south and east. A number of states from the northern, central and eastern parts of the country rank low in human development index and exhibit relatively poor nutritional and health circumstances as compared to other states from the southern and eastern parts of India with the exception of the north-eastern states. These geographic differences in the nutritional and health status of the population may partially bring about differentials in the age at menarche of women [71]. Across Indian states, women from Assam, Arunachal Pradesh, Sikkim, Karnataka and Andhra Pradesh recorded the lowest mean age at menarche. On the contrary, women from Himachal Pradesh, Uttaranchal, Haryana, Jammu and Kashmir and Jharkhand experienced the highest mean menarcheal age. In addition, this study also documented substantial variations in mean menarcheal age across major linguistic groups in India. Notably, most of the non-Hindi speaking women experienced early mean age at menarche when compared to their Hindi speaking counterparts. These spatio-cultural variations exhibit tremendous long standing genetic/ethnic diversity, dietary patterns and gender roles that might account for differences in mean menarcheal age across various geographic regions of India.

This study clearly brings out substantial socioeconomic patterning of mean age at menarche among Indian women. Interestingly, mean age at menarche was significantly higher among women with higher education, economically better of households and socially forward castes groups than their counterparts. Previous studies have arrived at mixed conclusions with regard to the association between socioeconomic status and mean age at menarche [72]. However, our result does not corroborate the findings of some of the earlier micro-level studies in India. It is possible that many women with a high socioeconomic status might be particular about the shape of their body/physical appearance and hence exercise/work out intensively, which may push their mean menarcheal age to upper thresholds [73]. We also found that women residing in urban areas experience a relatively early mean age at menarche compared to their rural counterparts. Our results are consistent with similar findings from China, where rural women have a relatively higher mean age at menarche than their urban counterparts [30]. Data also indicate significant variations in mean age at menarche across religious groups. Muslim women experienced significantly early mean age at menarche than their Hindu counterparts. However, our result does not support any significant association between body mass index and mean age at menarche of women in India.

To sum up, we report three take home messages that emerged from the present analyses. First, we observed secular declining trends in mean age at menarche among Indian women during 1955–1989. The findings have indicated a decline of one month per decade in the mean age at menarche in India. Second, there was glaring spatial heterogeneity in mean menarcheal age across birth cohorts of women in India. Lastly, the study highlighted sharp socioeconomic and contextual pattering in mean menarcheal age among women. Women who were better educated, economically well-off and from forward/general caste groups experienced a higher mean age at menarche. Women residing in urban areas, from north-eastern/southern/eastern regions, speaking non-Hindi languages experienced a relatively early mean age at menarche.

### Limitations of the study

Though the present study adds to the literature on menarcheal age in the Indian context. There are some limitations that need to be considered while interpreting the results. The measurement of the menarcheal age is based on the retrospective or recall method. There are some studies which criticize the recall method for over-reporting the menarcheal age, while certain studies have accused it for under-reporting the menarcheal age [74]–[76]. Some have compared recall data with other sources of information and noted that the recall method provides fairly consistent estimates [37].

Second, this study uses IHDS-2004/2005 data set which provides retrospective information. Hence any estimates generated from the present data will provide older reference dates compared to the recent status-quo studies. However, to tackle this issue by clubbing the study subjects into birth cohorts. Therefore, this comparative pseudo-cohort approach emerged as the potential strength of present study. Finally, given the cross sectional nature of the data, we could not identify any causal relationship between the outcome and exposure variables. Rather, we only identify the association between the salient socioeconomic, anthropometric and contextual characteristics with the menarcheal age of women. Therefore, we suggest that future studies take up large scale status-quo and longitudinal surveys to evaluate the present scenario and identify the causal risk factors of menarcheal age among women in India.

## Supporting Information

Figure S1 **Scree plot representing eigen values from Principal Component Analysis (PCA), India, IHDS, 2004–2005.**
(TIF)

Table S1 **Mean age at menarche by educational status of women (15–49y) across states in India, IHDS, 2004–2005.**
(DOCX)

Table S2 **Mean age at menarche by caste groups of women (15–49y) across states in India, IHDS, 2004–2005.**
(DOCX)

Table S3 **Mean age at menarche by wealth status of women (15–49y) across states in India, IHDS, 2004–2005.**
(DOCX)

Table S4 **Mean age at menarche by residence of women (15–49y) across states in India, IHDS, 2004–2005.**
(DOCX)
