# Supplementary figures and images for: Secular Trends in Menarcheal Age in India-Evidence from the Indian Human Development Survey
Source: PLoS One. 2014 Nov 4;9(11):e111027. doi: 10.1371/journal.pone.0111027 (PMC4219698; doi:10.1371/journal.pone.0111027)

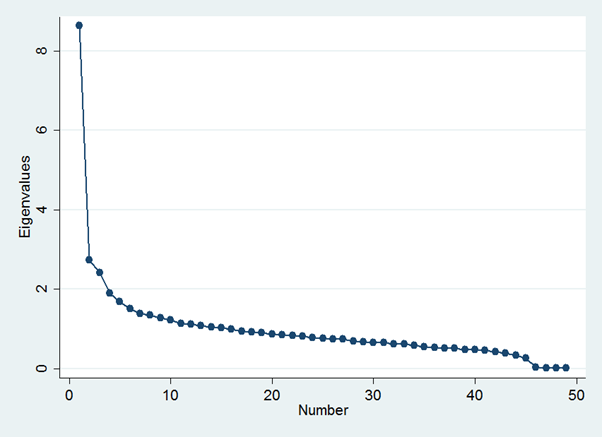

Supplement: Figure S1 — Scree plot representing eigen values from Principal Component Analysis (PCA), India, IHDS, 2004–2005. (TIF) [file pone.0111027.s001.tif]
