# Supplementary material for: Secular Trends in Menarcheal Age in India-Evidence from the Indian Human Development Survey
Source: PLoS One. 2014 Nov 4;9(11):e111027. doi: 10.1371/journal.pone.0111027 (PMC4219698; doi:10.1371/journal.pone.0111027)
Supplement: Table S1 — Mean age at menarche by educational status of women (15–49y) across states in India, IHDS, 2004–2005. (DOCX) [file pone.0111027.s002.docx]

|  | | | | | | | | | | | |
| --- | --- | --- | --- | --- | --- | --- | --- | --- | --- | --- | --- |
| **State** | **None** |  | **1-5y** |  | **6-10y** |  | **11-12y** |  | **>=13y** |  |  |
|  | *Age at menarche* | *S.D.* | *Age at menarche* | *S.D.* | *Age at menarche* | *S.D.* | *Age at menarche* | *S.D.* | *Age at menarche* | *S.D.* | *P-value* |
| J&K | 14.360 | 1.588 | 14.108 | 2.119 | 14.079 | 0.171 | 14.737 | 1.137 | 15.045 | 1.265 | 0.000 |
| HP | 14.931 | 1.471 | 15.098 | 1.361 | 15.239 | 1.475 | 15.163 | 1.606 | 14.486 | 1.381 | 0.000 |
| PJ | 14.050 | 1.088 | 14.127 | 1.082 | 14.390 | 0.956 | 14.176 | 0.720 | 14.264 | 0.856 | 0.000 |
| UT | 14.354 | 1.229 | 14.456 | 1.080 | 14.560 | 1.319 | 15.000 | 0.615 | 14.613 | 0.732 | 0.000 |
| HR | 14.298 | 1.375 | 14.640 | 1.220 | 14.546 | 1.246 | 14.682 | 1.193 | 15.041 | 1.066 | 0.000 |
| DL | 13.591 | 1.584 | 13.296 | 1.787 | 13.638 | 1.370 | 13.635 | 1.453 | 13.958 | 1.332 | 0.000 |
| RJ | 13.999 | 1.386 | 13.881 | 1.201 | 14.183 | 1.253 | 14.312 | 1.269 | 14.333 | 1.147 | 0.000 |
| UP | 14.054 | 1.022 | 14.160 | 1.070 | 14.229 | 1.057 | 14.222 | 1.152 | 14.094 | 1.222 | 0.000 |
| BH | 13.644 | 1.144 | 13.522 | 1.137 | 13.894 | 1.366 | 14.044 | 1.224 | 13.705 | 1.271 | 0.000 |
| SK | 12.000 | 0.292 | 12.000 | 0.000 | 12.065 | 0.247 | 12.000 | 0.000 | 12.000 | 0.000 | 0.398 |
| AR | 12.123 | 0.633 | 12.174 | 0.388 | 12.388 | 0.619 | 12.615 | 0.637 | 12.611 | 0.502 | 0.000 |
| NG | 14.091 | 1.044 | 13.375 | 1.025 | 12.732 | 1.114 | 13.786 | 1.424 | 14.000 | 0.000 | 0.000 |
| MN | 14.049 | 1.284 | 14.455 | 1.508 | 13.846 | 1.145 | 14.520 | 1.194 | 14.654 | 1.149 | 0.000 |
| MZ | 14.000 | 0.000 | 14.571 | 0.504 | 14.348 | 0.755 | 14.000 | 0.000 | 14.000 | 0.000 | 0.000 |
| TR | 13.391 | 0.901 | 13.456 | 0.766 | 13.335 | 1.023 | 14.091 | 0.831 | 13.688 | 1.014 | 0.000 |
| MG | 13.170 | 1.372 | 13.672 | 1.295 | 13.377 | 1.051 | 14.750 | 0.866 | 13.926 | 1.519 | 0.000 |
| AS | 12.016 | 0.937 | 11.872 | 1.099 | 11.895 | 0.968 | 11.864 | 0.991 | 12.351 | 0.633 | 0.009 |
| WB | 13.467 | 1.329 | 13.246 | 1.303 | 13.153 | 1.244 | 13.223 | 1.563 | 12.973 | 1.122 | 0.000 |
| JH | 13.791 | 1.131 | 13.639 | 1.136 | 13.967 | 1.128 | 13.769 | 0.815 | 14.217 | 1.451 | 0.000 |
| OD | 13.184 | 0.751 | 13.266 | 0.902 | 13.415 | 1.026 | 13.925 | 1.039 | 13.540 | 1.048 | 0.000 |
| CHH | 13.849 | 0.933 | 13.992 | 1.125 | 14.093 | 1.008 | 14.336 | 0.922 | 14.103 | 1.046 | 0.000 |
| MP | 13.900 | 0.965 | 13.893 | 0.897 | 14.178 | 1.101 | 13.834 | 0.848 | 14.300 | 0.959 | 0.000 |
| GJ | 13.737 | 0.889 | 13.902 | 1.234 | 13.896 | 1.084 | 14.083 | 1.013 | 14.063 | 1.207 | 0.000 |
| MH | 14.125 | 1.197 | 13.972 | 1.074 | 14.222 | 1.163 | 14.468 | 1.122 | 14.396 | 1.180 | 0.000 |
| AP | 13.099 | 1.104 | 12.985 | 0.991 | 13.320 | 1.234 | 13.873 | 1.381 | 13.770 | 0.938 | 0.000 |
| KN | 12.756 | 1.088 | 12.787 | 1.126 | 13.130 | 1.375 | 13.501 | 1.481 | 13.689 | 1.597 | 0.000 |
| Goa | 13.304 | 1.396 | 12.694 | 1.262 | 13.127 | 1.080 | 13.095 | 1.358 | 14.132 | 2.849 | 0.000 |
| KR | 13.588 | 1.641 | 13.283 | 1.089 | 13.317 | 1.253 | 13.064 | 1.213 | 13.278 | 1.137 | 0.000 |
| TN | 13.789 | 1.343 | 13.765 | 1.220 | 13.856 | 1.185 | 14.035 | 1.237 | 14.108 | 1.237 | 0.000 |
| Note: S.D. refers to standard deviation; Analysis of variance test used to examine differences in mean age at menarche across educational groups of women; Abbreviation used for states of India: J&K- Jammu and Kashmir, HP- Himachal Pradesh, PJ- Punjab, UT- Uttarakhand, HR-Haryana, DL- Delhi, RJ- Rajasthan, UP- Uttar Pradesh, BH- Bihar, SK- Sikkim, AR- Arunachal Pradesh, NG- Nagaland, MN- Manipur, MZ- Mizoram, TR- Tripura, MG-Meghalaya, AS-Assam, WB- West Bengal, JH- Jharkhand, OD- Odisha, CHH- Chhattisgarh, MP- Madhya Pradesh, GJ- Gujarat, MH- Maharashtra, AP- Andhra Pradesh, KN- Karnataka, KR- Kerala, TN- Tamil Nadu. | | | | | | | | | | | |
