# Supplementary material for: Secular Trends in Menarcheal Age in India-Evidence from the Indian Human Development Survey
Source: PLoS One. 2014 Nov 4;9(11):e111027. doi: 10.1371/journal.pone.0111027 (PMC4219698; doi:10.1371/journal.pone.0111027)
Supplement: Table S2 — Mean age at menarche by caste groups of women (15–49y) across states in India, IHDS, 2004–2005. (DOCX) [file pone.0111027.s003.docx]

|  | | | | | | | | | |
| --- | --- | --- | --- | --- | --- | --- | --- | --- | --- |
| **State** | **SC** |  | **ST** |  | **OBC** |  | **FC** |  |  |
|  | *Age at menarche* | *S.D.* | *Age at menarche* | *S.D.* | *Age at menarche* | *S.D.* | *Age at menarche* | *S.D.* | *P-value* |
| J&K | 14.516 | 1.849 | 14.829 | 1.294 | 14.000 | 1.500 | 14.333 | 1.677 | 0.025 |
| HP | 14.892 | 1.417 | 15.098 | 1.154 | 15.175 | 1.467 | 15.154 | 1.502 | 0.000 |
| PJ | 14.126 | 1.039 | 14.000 | 0.000 | 14.265 | 1.085 | 14.235 | 0.969 | 0.007 |
| UT | 14.335 | 1.154 | 14.667 | 0.488 | 14.146 | 1.217 | 14.793 | 1.117 | 0.000 |
| HR | 14.395 | 1.328 | 13.912 | 1.083 | 14.351 | 1.480 | 14.591 | 1.143 | 0.000 |
| DL | 13.486 | 1.477 | 12.667 | 1.803 | 13.431 | 1.429 | 13.767 | 1.584 | 0.000 |
| RJ | 13.718 | 1.350 | 14.048 | 1.214 | 14.129 | 1.349 | 14.145 | 1.305 | 0.000 |
| UP | 14.120 | 0.984 | 14.183 | 0.850 | 14.106 | 1.060 | 14.090 | 1.080 | 0.718 |
| BH | 13.533 | 1.276 | 13.500 | 0.894 | 13.723 | 1.138 | 13.778 | 1.322 | 0.000 |
| SK | 12.065 | 0.247 | 12.000 | 0.000 | 12.000 | 0.000 | 12.000 | 0.256 | 0.254 |
| AR | 12.000 | 0.000 | 12.267 | 0.590 | 13.000 | 0.000 | 12.077 | 1.017 | 0.008 |
| NG | na | na | 13.072 | 1.232 | 13.111 | 1.167 | 12.167 | 0.408 | 0.201 |
| MN | na | na | na | na | 14.141 | 1.234 | 14.350 | 0.671 | 0.000 |
| MZ | na | na | 14.305 | 0.640 | na | na | na | na | na |
| TR | 13.438 | 1.029 | 13.536 | 0.613 | 13.360 | 0.969 | 13.358 | 1.022 | 0.478 |
| MG | 13.333 | 0.500 | 13.527 | 1.350 | 13.480 | 1.122 | 14.260 | 1.337 | 0.004 |
| AS | 11.766 | 1.146 | 11.790 | 0.799 | 12.063 | 1.087 | 11.936 | 0.939 | 0.001 |
| WB | 13.374 | 1.428 | 13.718 | 1.117 | 13.308 | 1.221 | 13.199 | 1.249 | 0.000 |
| JH | 13.579 | 1.004 | 13.778 | 1.300 | 13.826 | 1.067 | 14.119 | 1.147 | 0.000 |
| OD | 13.178 | 0.956 | 13.319 | 0.837 | 13.329 | 0.868 | 13.339 | 0.936 | 0.000 |
| CHH | 14.140 | 1.184 | 13.770 | 0.760 | 13.977 | 1.050 | 14.025 | 0.934 | 0.000 |
| MP | 13.804 | 0.969 | 14.000 | 0.974 | 13.917 | 0.973 | 14.102 | 0.959 | 0.000 |
| GJ | 13.983 | 1.325 | 13.777 | 0.799 | 13.778 | 0.998 | 13.914 | 1.040 | 0.000 |
| MH | 13.986 | 1.176 | 14.064 | 1.193 | 14.162 | 1.167 | 14.276 | 1.134 | 0.000 |
| AP | 13.036 | 1.083 | 12.799 | 0.979 | 13.200 | 1.146 | 13.324 | 1.187 | 0.000 |
| KN | 12.776 | 1.185 | 12.896 | 1.087 | 12.953 | 1.217 | 13.204 | 1.534 | 0.000 |
| Goa | 12.167 | 0.408 | na | na | 13.116 | 1.036 | 13.148 | 1.323 | 0.000 |
| KR | 13.235 | 1.259 | 14.125 | 2.335 | 13.231 | 1.238 | 13.324 | 1.191 | 0.007 |
| TN | 13.633 | 1.267 | 13.297 | 1.102 | 13.978 | 1.242 | 13.456 | 1.107 | 0.000 |
| Note: S.D. refers to standard deviation; Analysis of variance test used to examine differences in mean age at menarche across caste groups of women; na indicate data not available; Abbreviation used for states of India: J&K- Jammu and Kashmir, HP- Himachal Pradesh, PJ- Punjab, UT- Uttarakhand, HR-Haryana, DL- Delhi, RJ- Rajasthan, UP- Uttar Pradesh, BH- Bihar, SK- Sikkim, AR- Arunachal Pradesh, NG- Nagaland, MN- Manipur, MZ- Mizoram, TR- Tripura, MG-Meghalaya, AS-Assam, WB- West Bengal, JH- Jharkhand, OD- Odisha, CHH- Chhattisgarh, MP- Madhya Pradesh, GJ- Gujarat, MH- Maharashtra, AP- Andhra Pradesh, KN- Karnataka, KR- Kerala, TN- Tamil Nadu. | | | | | | | | | |
