# Supplementary material for: Secular Trends in Menarcheal Age in India-Evidence from the Indian Human Development Survey
Source: PLoS One. 2014 Nov 4;9(11):e111027. doi: 10.1371/journal.pone.0111027 (PMC4219698; doi:10.1371/journal.pone.0111027)
Supplement: Table S3 — Mean age at menarche by wealth status of women (15–49y) across states in India, IHDS, 2004–2005. (DOCX) [file pone.0111027.s004.docx]

|  | | | | | | | | | | | |
| --- | --- | --- | --- | --- | --- | --- | --- | --- | --- | --- | --- |
| **State** | **Poorest** |  | **Poorer** |  | **Middle** |  | **Richer** |  | **Richest** |  |  |
|  | ***Age at menarche*** | ***S.D.*** | ***Age at menarche*** | ***S.D.*** | ***Age at menarche*** | ***S.D.*** | ***Age at menarche*** | ***S.D.*** | ***Age at menarche*** | ***S.D.*** | ***P-value*** |
| J&K | 14.288 | 2.051 | 14.260 | 1.462 | 14.519 | 1.765 | 14.273 | 1.731 | 14.179 | 1.523 | 0.024 |
| HP | 14.583 | 0.515 | 14.809 | 1.375 | 15.305 | 1.498 | 15.104 | 1.472 | 14.904 | 1.426 | 0.000 |
| PJ | 13.786 | 1.260 | 13.885 | 1.181 | 14.119 | 1.085 | 14.151 | 1.036 | 14.364 | 0.896 | 0.000 |
| UT | 14.363 | 1.265 | 14.285 | 1.170 | 14.388 | 1.309 | 14.646 | 1.114 | 14.546 | 1.010 | 0.006 |
| HR | 14.347 | 1.058 | 14.328 | 1.320 | 14.207 | 1.372 | 14.500 | 1.379 | 14.750 | 1.160 | 0.000 |
| DL | 14.500 | 1.732 | 13.073 | 1.766 | 13.421 | 1.772 | 13.531 | 1.531 | 13.664 | 1.475 | 0.026 |
| RJ | 13.909 | 1.319 | 13.800 | 1.301 | 14.168 | 1.500 | 14.163 | 1.197 | 14.262 | 1.306 | 0.000 |
| UP | 14.123 | 1.063 | 13.974 | 0.957 | 14.192 | 0.994 | 14.120 | 1.151 | 14.134 | 1.079 | 0.000 |
| BH | 13.662 | 1.129 | 13.829 | 1.146 | 13.866 | 1.375 | 13.494 | 1.343 | 13.568 | 1.241 | 0.000 |
| SK | na | na | na | na | 12.000 | 0.000 | 12.026 | 0.228 | 12.091 | 0.294 | 0.357 |
| AR | na | na | 12.167 | 0.408 | 12.069 | 0.536 | 12.430 | 0.728 | 12.394 | 0.551 | 0.000 |
| NG | na | na | 13.000 | 0.000 | 13.311 | 1.145 | 12.966 | 1.254 | 12.000 | 0.000 | 0.071 |
| MN | 13.000 | 0.000 | 14.250 | 1.357 | 14.314 | 1.407 | 14.126 | 1.429 | 14.267 | 0.747 | 0.146 |
| MZ | na | na | 14.000 | 0.000 | 15.286 | 0.756 | 14.458 | 0.768 | 14.069 | 0.256 | 0.000 |
| TR | 13.517 | 0.738 | 13.705 | 0.887 | 13.156 | 0.927 | 13.520 | 0.893 | 13.029 | 0.969 | 0.000 |
| MG | 12.833 | 1.403 | 13.574 | 1.291 | 13.384 | 1.036 | 13.351 | 1.670 | 14.106 | 1.324 | 0.000 |
| AS | 11.750 | 1.355 | 12.105 | 0.962 | 12.078 | 0.678 | 11.720 | 1.121 | 11.614 | 1.127 | 0.000 |
| WB | 13.440 | 1.288 | 13.257 | 1.294 | 13.357 | 1.408 | 13.117 | 1.277 | 13.112 | 1.225 | 0.000 |
| JH | 13.741 | 1.234 | 13.905 | 1.188 | 13.700 | 0.918 | 13.846 | 0.954 | 14.054 | 1.192 | 0.000 |
| OD | 13.188 | 0.780 | 13.318 | 0.935 | 13.327 | 0.777 | 13.411 | 0.972 | 13.504 | 1.174 | 0.000 |
| CHH | 13.828 | 0.942 | 13.919 | 0.979 | 14.022 | 0.963 | 14.345 | 1.308 | 14.168 | 1.011 | 0.000 |
| MP | 13.848 | 1.042 | 13.882 | 0.906 | 14.057 | 1.007 | 14.026 | 0.844 | 14.151 | 0.965 | 0.000 |
| GJ | 13.864 | 0.816 | 13.707 | 0.874 | 13.723 | 0.928 | 13.867 | 1.115 | 14.026 | 1.186 | 0.000 |
| MH | 14.227 | 1.134 | 14.197 | 1.204 | 14.209 | 1.155 | 14.170 | 1.151 | 14.090 | 1.156 | 0.016 |
| AP | 12.862 | 0.992 | 13.145 | 1.116 | 13.145 | 1.200 | 13.143 | 1.099 | 13.490 | 1.125 | 0.000 |
| KN | 12.682 | 1.099 | 12.762 | 1.159 | 12.880 | 1.112 | 13.105 | 1.250 | 13.448 | 1.564 | 0.000 |
| Goa | na | na | 12.500 | 2.121 | 12.708 | 1.233 | 13.109 | 1.305 | 13.265 | 1.559 | 0.280 |
| KR | 13.000 | 0.795 | 13.393 | 1.410 | 13.437 | 1.244 | 13.208 | 1.237 | 13.257 | 1.188 | 0.001 |
| TN | 13.680 | 1.463 | 13.738 | 1.253 | 13.873 | 1.218 | 13.870 | 1.232 | 13.934 | 1.248 | 0.009 |
| Note: S.D. refers to standard deviation; Analysis of variance test used to examine differences in mean age at menarche across wealth groups of women; na indicate data not available; Abbreviation used for states of India: J&K- Jammu and Kashmir, HP- Himachal Pradesh, PJ- Punjab, UT- Uttarakhand, HR-Haryana, DL- Delhi, RJ- Rajasthan, UP- Uttar Pradesh, BH- Bihar, SK- Sikkim, AR- Arunachal Pradesh, NG- Nagaland, MN- Manipur, MZ- Mizoram, TR- Tripura, MG-Meghalaya, AS-Assam, WB- West Bengal, JH- Jharkhand, OD- Odisha, CHH- Chhattisgarh, MP- Madhya Pradesh, GJ- Gujarat, MH- Maharashtra, AP- Andhra Pradesh, KN- Karnataka, KR- Kerala, TN- Tamil Nadu. | | | | | | | | | | | |
