# Supplementary material for: Secular Trends in Menarcheal Age in India-Evidence from the Indian Human Development Survey
Source: PLoS One. 2014 Nov 4;9(11):e111027. doi: 10.1371/journal.pone.0111027 (PMC4219698; doi:10.1371/journal.pone.0111027)
Supplement: Table S4 — Mean age at menarche by residence of women (15–49y) across states in India, IHDS, 2004–2005. (DOCX) [file pone.0111027.s005.docx]

|  | | | | | |
| --- | --- | --- | --- | --- | --- |
| **State** | **Rural** |  | **Urban** |  |  |
|  | ***Age at menarche*** | ***S.D.*** | ***Age at menarche*** | ***S.D.*** | ***P-value*** |
| J&K | 14.426 | 1.685 | 14.187 | 1.649 | 0.005 |
| HP | 15.152 | 1.435 | 14.813 | 1.559 | 0.000 |
| PJ | 14.218 | 1.067 | 14.178 | 0.906 | 0.267 |
| UT | 14.429 | 1.234 | 14.565 | 1.008 | 0.104 |
| HR | 14.422 | 1.340 | 14.631 | 1.150 | 0.001 |
| DL | 13.109 | 1.602 | 13.637 | 1.513 | 0.000 |
| RJ | 14.043 | 1.396 | 14.005 | 1.217 | 0.310 |
| UP | 14.097 | 1.064 | 14.121 | 1.018 | 0.311 |
| BH | 13.738 | 1.124 | 13.599 | 1.360 | 0.001 |
| SK | 12.055 | 0.229 | 12.000 | 0.214 | 0.086 |
| AR | 12.223 | 0.671 | 12.369 | 0.485 | 0.067 |
| NG | 13.219 | 1.260 | 12.231 | 0.430 | 0.000 |
| MN | 14.091 | 1.391 | 14.353 | 0.875 | 0.087 |
| MZ | 14.584 | 0.801 | 14.026 | 0.160 | 0.000 |
| TR | 13.604 | 0.826 | 12.671 | 0.944 | 0.000 |
| MG | 13.416 | 1.187 | 13.983 | 1.489 | 0.000 |
| AS | 12.029 | 0.878 | 11.722 | 1.179 | 0.000 |
| WB | 13.387 | 1.291 | 13.143 | 1.316 | 0.000 |
| JH | 13.800 | 1.180 | 13.895 | 1.085 | 0.053 |
| OD | 13.264 | 0.839 | 13.379 | 1.030 | 0.000 |
| CHH | 13.882 | 0.923 | 14.184 | 1.195 | 0.000 |
| MP | 13.917 | 1.004 | 14.041 | 0.856 | 0.000 |
| GJ | 13.783 | 0.888 | 13.953 | 1.225 | 0.000 |
| MH | 14.217 | 1.134 | 14.092 | 1.208 | 0.000 |
| AP | 13.201 | 1.121 | 13.154 | 1.181 | 0.169 |
| KN | 12.866 | 1.167 | 13.174 | 1.449 | 0.000 |
| Goa | 12.991 | 1.252 | 13.489 | 1.749 | 0.002 |
| KR | 13.372 | 1.184 | 13.121 | 1.285 | 0.000 |
| TN | 13.900 | 1.248 | 13.812 | 1.259 | 0.043 |
| Note: S.D. refers to standard deviation; Analysis of variance test used to examine differences in mean age at menarche across residence of women; Abbreviation used for states of India: J&K- Jammu and Kashmir, HP- Himachal Pradesh, PJ- Punjab, UT- Uttarakhand, HR-Haryana, DL- Delhi, RJ- Rajasthan, UP- Uttar Pradesh, BH- Bihar, SK- Sikkim, AR- Arunachal Pradesh, NG- Nagaland, MN- Manipur, MZ- Mizoram, TR- Tripura, MG-Meghalaya, AS-Assam, WB- West Bengal, JH- Jharkhand, OD- Odisha, CHH- Chhattisgarh, MP- Madhya Pradesh, GJ- Gujarat, MH- Maharashtra, AP- Andhra Pradesh, KN- Karnataka, KR- Kerala, TN- Tamil Nadu. | | | | | |
